# POTENTIAL MECHANISMS THROUGH WHICH INDUCING HEART RATE OSCILLATIONS AFFECTS PLASMA AMYLOID BETA LEVELS

**DOI:** 10.1093/geroni/igad104.0219

**Published:** 2023-12-21

**Authors:** Jungwon Min, Jeremy Rouanet, Steve Cole, Kaoru Nashiro, Hyun Joo Yoo, Christine Cho, Elizabeth Head, Mara Mather

**Affiliations:** University of Southern California, Los Angeles, California, United States; University of California, Irvine, Irvine, California, United States; University of California Los Angeles, Los Angeles, California, United States; University of Southern California, Los Angeles, California, United States; University of Southern California, Los Angeles, California, United States; University of Southern California, Los Angeles, California, United States; University of California, Irvine, Irvine, California, United States; University of Southern California, Los Angeles, California, United States

## Abstract

Alzheimer’s disease-related protein amyloid beta (Aβ) plasma levels increase with age, and Aβ40 levels are associated with greater mortality. Most plasma Aβ40 is produced via platelets. Platelet activity is regulated by endothelial cells, which are stimulated by blood flow. Slow paced breathing with a 10-second cycle maximizes heart rate oscillations, which may stimulate endothelial-platelet interactions that affect Aβ production. We investigated this idea by randomizing healthy adults (54 younger and 54 older adults) into two groups with the opposite goals: increasing (Osc+) or decreasing (Osc-) heart rate oscillations. Four weeks of daily practice (20-40 min) resulted in large changes in plasma Aβ40 and Aβ42 levels. We also found that changes in Aβ were not associated with changes in resting-state physiology including heart rate variability. But the Aβ changes were associated with a frequency measure of heart rate oscillations induced during practice, suggesting that the Aβ changes were more driven by blood flow changes during practice. P-selectin is an indicator of endothelial activity. We found a significant intervention effect where the Osc+ intervention increased p-selectin and the Osc- intervention decreased p-selectin in older adults. Changes in p-selectin also showed strong negative correlations with changes in Aβ40 and Aβ42 in older adults. The results suggest that blood flow oscillations caused by high amplitude heart rate oscillations stimulated endothelial cells, which suppressed platelet activity and consequently reduced the production of Aβ in older adults. In follow-up research, we are investigating relationships between plasma Ab changes and changes in brain and cognition.

